# Potential role of serological biomarkers in the diagnosis and prediction of community-acquired pneumonia in elderly individuals

**DOI:** 10.3389/fmed.2025.1699779

**Published:** 2025-11-20

**Authors:** Chen Hou, Yi Feng Jin, Jie Li

**Affiliations:** Department of General Practice, The First Affiliated Hospital of Soochow University, Suzhou, China

**Keywords:** community-acquired pneumonia (CAP), elderly patients, serological biomarkers, diagnosis, prognosis

## Abstract

Community-acquired pneumonia (CAP) is one of the most common lower respiratory tract infections (LRTIs) and has substantial clinical and economic impacts on healthcare systems worldwide. Because of impaired host defenses and/or underlying health problems, CAP in elderly patients is associated with increased mortality and morbidity compared with that in younger patients and is prone to developing severe community-acquired pneumonia (SCAP). Diagnosis, severity evaluation, and prognosis are still challenges for physicians. Therefore, new diagnostic methods are needed to identify the different stages of CAP and monitor disease progression. The value of serological biomarkers has been extensively investigated in infectious diseases. In clinical practice, there are currently no defined or agreed-upon biomarker(s) for CAP that can be readily tested. An ideal biomarker that is simple, easy to perform, noninvasive or microinvasive, inexpensive, rapid, and reproducible is helpful for patients and clinicians. The aim of this review is to highlight potential serological biomarkers for the diagnosis and prediction of CAP in elderly individuals, providing novel strategies for patient stratification and treatment.

## Introduction

Community-acquired pneumonia (CAP) remains the leading cause of mortality and morbidity worldwide. Several factors such as geographical region, age and study period influence the incidence of CAP in adults (1, 2). The number of deaths from lower respiratory tract infections (LRTIs) is nearly 2.4 million in all age groups, including more than 1.08 million elderly patients over 70 years old (3). Despite important advances in prevention through vaccines, new rapid diagnostic tests and antibiotics, mortality due to LRTI remained unchanged from 2005 to 2015, although age-standardized death rates decreased by 19.5%, and a parallel improvement in the lower respiratory infection mortality rate among elderly individuals is generally not observed (4). CAP in elderly patients is prone to respiratory failure or shock and progression to severe community-acquired pneumonia (SCAP) (5, 6). Therefore, early recognition of severe disease, and associated identification of patients at increased risk of mortality who require intensive monitoring and/or intensive care are highly important.

Biomarkers can be objectively measured and assessed as biological indicators of normal physiological processes, pathological conditions, or pharmacological responses to therapeutic interventions. These biomarkers, which are present in blood, other biological fluids, and tissues, can indicate the presence of abnormal states or diseases within the body (7). Recent studies have demonstrated their significant role in evaluating both the severity and treatment efficacy of CAP, particularly in relation to patient mortality (8, 9). Although the specific pathogenesis of CAP remains incompletely understood, it is widely accepted that it arises from pathogenic infections that elicit an aberrant immune response in the host. Upon invasion by pathogens—such as bacteria and viruses—the immune system is activated, orchestrating the release of various proinflammatory cytokines and acute-phase proteins (10, 11). While this proinflammatory response is critical for pathogen elimination, excessive inflammation can result in detrimental effects for the host, including shock, coagulopathy, and tissue damage, ultimately leading to organ dysfunction and death. Furthermore, the immune response during infection involves an anti-inflammatory component, which is vital for restoring homeostasis and mitigating collateral damage (12–14). Thus, stratification on the basis of biomarkers is essential. The overactivation of inflammatory responses and the resulting tissue damage are pivotal in the onset and progression of CAP (12). A variety of inflammatory mediators are implicated in the pathogenesis of CAP, offering potential value in facilitating diagnosis and reflecting the extent of inflammation within the body.

Given the uncertainty about the precise role of biomarkers in the management of elderly patients with community-acquired pneumonia (CAP), we reviewed the literature on serological biomarkers used in clinical and experimental studies to better evaluate their utility. We explicitly listed the database searched (PubMed) and reiterated the search period (2010–2025). The full PubMed search strategy is provided in the main text and includes all subject headings, free-text terms, and Boolean operators used. Complete search strategies for the other databases are included in the Supplementary Appendix 1 to ensure transparency and reproducibility. In addition to database searches, we manually screened the reference lists of included studies to identify any potentially relevant articles, thereby enhancing the comprehensiveness of the literature search. Studies of biomarkers in elderly patients with CAP are summarized in Table 1.

**TABLE 1:** Summary of biomarkers for biological markers in elderly patients with community-acquired pneumonia.

Predictive scenario and biomarker	Sample composition	Risk estimate (OR/HR)	*P*-value	Discrimination (AUC/C-statistic)	Clinical cutoff and remarks	References
**Part A: Immune status and general prognosis**						
Total early-differentiated T-cells	96 elderly patients	OR = 6.3	< 0.05	0.796	Predicts 2-month mortality; Cutoff < 433 cells/μL.	(15)
CD4+ early-differentiated T-cells	96 elderly patients	OR = 4.8 OR = 2.6	< 0.05	0.713 0.642	Predicts 1-year mortality (Cutoff < 505 cells/μL). Predicts hospital readmission (Cutoff < 532 cells/μL).	(15)
**Part B: CAP severity and short-term prognosis**						
lncRNA TUG1 (low expression)	130 sCAP vs. 122 controls	OR ≈ 2.65	< 0.01	0.823	Predicts 30-day mortality; high PPV (83.3%).	(16)
BNP	158 CAP patients	OR = 1.003	0.001	0.803	Assesses CAP severity, potentially reflecting cardiac involvement.	(17)
CRP	158 CAP patients	OR = 1.011	0.039	0.791	Conventional inflammation marker for severity assessment.	(18)
D-dimer	158 CAP patients	OR = 1.289	0.039	0.727	Predict pneumonia severity in elderly CAP patients	
NLR (Neutrophil-to-Lymphocyte Ratio)	158 CAP patients	OR = 1.111	0.039	0.817	Predicts CAP severity with good discrimination.	
MPI (at admission)	50 CAP patients	HR = 1.42	0.014	0.667	Predicts all-cause mortality.	
MPI (at discharge)	50 CAP patients	HR = 2.45	< 0.001	0.851	Predicts all-cause mortality with better discrimination than MPI at admission	(19)
pro-ADM (admission, D1, D3, D5)	50 CAP patients	HR = 1.14–1.70	< 0.05	0.670–0.744	Dynamic monitoring; risk increases over time (highest HR on day 5).	
MPI (admission) + pro-ADM (admission)	50 CAP patients	N/A	0.018	0.667 → 0.731	Significantly improves all-cause mortality prediction.	
MPI (admission) + PCT	49 CAP patients	N/A	0.020	0.65 → 0.69	Significantly improves 1-month mortality prediction (IDI = 0.045).	
**Part C: Muscle mass and long-term prognosis**						
Low muscle mass (Cr/CysC × 100)	606 elderly CAP patients	HR = 1.90 (1-year) HR = 1.85 (3-year)	< 0.001	N/A	Strong predictor of 1- to 3-year mortality. 1-year mortality: 56.3% vs. 31.9%.	(20)
Low muscle mass (AST/ALT)	606 elderly CAP patients	HR = 1.46 (1-year) HR = 1.35 (3-year)	< 0.001	N/A	Effective predictor of 1- to 3-year mortality. 1-year mortality: 51.2% vs. 37.0%.	(21)
Erector spinae muscle cross-sectional area (ESMcsa/BSA)	689 elderly pneumonia patients	N/A	< 0.001*^b^*	N/A	Independent predictor of in-hospital mortality. Lower values are associated with poorer survival.	

ADM, adrenomedullin; ALT, alanine aminotransferase; AST, aspartate aminotransferase; AUC, area under the curve; BNP, B-type natriuretic peptide; BSA, body surface area; CAP, community-acquired pneumonia; Cr, creatinine; CRP, C-reactive protein; CysC, cystatin C; HR, hazard ratio; IDI, integrated discrimination improvement; lncRNA, long non-coding RNA; MPI, multidimensional prognostic index; NLR, neutrophil-to-lymphocyte ratio; N/A, not available; OR, odds ratio; PCT, procalcitonin; PPV, positive predictive value; sCAP, severe community-acquired pneumonia.

*^b^P*-value for association with survival.

In recent years, the rapid advancement of testing technologies has led to the prominence of several novel biological markers. Significant progress has been made in the research of biomarkers for patients with CAP. The identification of these serum biomarkers primarily arises from a comprehensive investigation into the pathogenesis of CAP. This research aims to elucidate the relevant molecular pathways and mechanisms, thereby identifying potential serological biomarkers that are closely associated with the disease state.

## Genome–RNA

According to genomic research, certain genetic differences may have a major impact on an individual’s immune response and susceptibility to infection, which is why they are important in the onset and progression of inflammatory illnesses (22, 23). This is especially crucial for research on older people with CAP, since their prognosis is usually worse and they are more susceptible to the disease. In this context, researchers have focused on circular RNAs (circRNAs), a unique class of RNA molecules, which have emerged as a significant area of investigation because of their ability to regulate microRNA (miRNA) activity and interact with RNA-binding proteins, thus profoundly influencing gene expression (24). In their study, Zhao et al. (25) examined the diagnostic utility of four specific circRNAs in adult patients with CAP and revealed that these circRNAs exhibited high specificity and sensitivity. Notably, HSA_circ_0026579 demonstrated remarkable efficacy in differentiating viral pneumonia from nonviral pneumonia, including bacterial and mixed infections (25). Furthermore, in CAP patients, the expression profile of circRNAs varies according to the pathogenic agent involved, thereby potentially modulating the production of various inflammatory mediators and enhancing the immune response of the host to different pathogens (26, 27). Moreover, these circRNAs may regulate inflammatory pathways through the inhibition of particular miRNAs, which in turn promotes the activity of immune cells, such as macrophages and neutrophils. This enhancement bolsters their capacity to phagocytose bacteria and mitigate viral replication and expression (28, 29). Consequently, circRNAs may play a pivotal role in distinguishing infections from different pathogens in CAP patients, suggesting significant implications for identifying the etiology of CAP. Additionally, the circular long noncoding RNA TUG1 is considered a valuable biomarker for predicting the 30-day mortality rate in elderly patients with CAP (16). TUG1 is involved in the inflammatory process by regulating gene expression and influencing cellular signaling pathways. Its downregulation may facilitate the release of inflammatory factors, such as tumor necrosis factor α and interleukins, thereby further initiating an inflammatory cascade and contributing to the development of SCAP (16, 30, 31).

## Metabolome-lipid

Lipidomic research on lipidomic continually emphasizes the importance of lipid metabolism in the pathogenesis of CAP. A detailed analysis of the serum lipid profiles of CAP patients revealed that changes in certain specific lipid molecules are closely related to inflammatory responses and disease severity (32, 33). These lipids not only participate in cellular signaling and influence intercellular communication but also may regulate immune system function, thereby playing a crucial role in the development of CAP. Lipids are the principal constituents of pulmonary surfactant, which plays a critical role in reducing alveolar surface tension and preventing alveolar collapse (34). This mechanism is vital for maintaining normal respiratory function. In patients with CAP, the functionality of pulmonary surfactant may be compromised, leading to significant alterations in lipid metabolism. Furthermore, during the course of infection by various pathogens, the levels of lipid metabolites undergo differential changes (35). A study demonstrated that 13 specific lipid metabolites could effectively differentiate CAP patients from noninfected individuals, those with extrapulmonary infections, and patients suffering from non-CAP respiratory infections. Among these metabolites, lysophosphatidylcholine (LPC) has high specificity and sensitivity. LPCs are known to influence the activation and functionality of various immune cells, including macrophages and lymphocytes. Specifically, LPC enhances the inflammatory response by activating distinct cell signaling pathways and promoting the release of inflammatory mediators (36, 37). Notably, the changes in LPC levels during viral infections may differ from those observed in bacterial infections, indicating a nuanced role in the inflammatory response (38–40). This finding is consistent with a previously mentioned study on 13 lipid metabolites in elderly patients with CAP. Additionally, other investigations into respiratory infections have revealed that LPC levels may decrease in patients with viral infections, whereas they tend to remain elevated in patients with bacterial infection (41–43). Furthermore, LPC plays a crucial role in distinguishing infectious pathogens in patients with CAP in addition to exhibiting significant prognostic value for elderly patients with CAP. Research indicates that lower levels of LPC are significantly associated with the 30-day mortality rate in this demographic population (44). These findings suggest that LPC may function as a valuable biomarker, offering critical insights for clinicians in evaluating patient prognosis and developing appropriate treatment strategies. Hence, further investigation into the role of LPC in the context of elderly CAP patients may enhance our understanding of its potential as a prognostic indicator and aid in improving clinical outcomes.

## Cell counting

Cell counts play an important role in assessing the inflammatory response of the host to infection. Specifically, an increase in neutrophils is usually indicative of an acute bacterial infection, whereas changes in lymphocytes may reflect the effects of viral infection or chronic disease (45, 46). By monitoring the proportions of different immune cells, clinicians are able to gain a deeper understanding of the nature of the infection and its underlying mechanisms (47). Among these, the NLR is widely recognized as a simple, reliable, cost-effective severity parameter for evaluating critically ill patients across various stress events, such as peritonitis, abdominal sepsis, postoperative complications, severe sepsis, and septic shock. Research has shown that a high NLR serves as an independent predictor of prognosis in various clinical conditions, including sepsis, chronic obstructive pulmonary disease, acute pulmonary embolism, and cancer (48–53). A study conducted by Cataudella et al. (54) further confirmed that the NLR is important not only for assessing the condition of elderly CAP patients but also for effectively predicting adverse outcomes. In a cohort of 195 elderly patients hospitalized due to CAP, an increase in the NLR was associated with a corresponding increase in mortality rates. When the NLR ranged from 13.4 to 28.3, the 30-day mortality rate for patients reached 50%, whereas all patients with an NLR greater than 28.3 died within 30 days. These findings suggest that the NLR could serve as an important prognostic biomarker for elderly CAP patients, with higher NLR values typically indicating a poorer prognosis. The study conducted by Bian et al. (55) provides a new perspective and hope for exploring the application of biological markers in elderly CAP patients. This study specifically focused on 15 elderly patients with CAP and investigated whether there is an imbalance in the number of regulatory T-cell (Treg) populations in their bodies. The results revealed that both the number of CD4+ T cells and the number of factors related to CD4+ T cells were significantly lower than those in the matched healthy control groups. Furthermore, the response of regulatory T cells is closely related to the severity of the disease (56). This finding prompted further consideration of regulatory T-cell-related factors, especially regarding whether examining changes in these factors could provide more information for assessing the severity of CAP in elderly patients. In particular, the characteristic factor of regulatory T cells, FOXP3 (a gene that encodes a transcription factor that is expressed primarily in Tregs and serves as a hallmark of Tregs), is especially important in this field (57). The expression level of FOXP3 is directly related to the development and function of regulatory T cells, and its dysregulation may lead to immune response disorders, thereby exacerbating the condition of elderly patients with CAP. Additionally, interleukin-10 (IL-10), a crucial anti-inflammatory cytokine that promotes the development and function of Tregs, also plays an indispensable role in this process (58). A lack of IL-10 may lead to persistent and exacerbated inflammatory responses, affecting patient prognosis. Notably, perforin, a key protein produced by immune system cells, participates in specific cytotoxic responses and helps regulate and eliminate abnormal or infected cells. The activity of perforin is closely related to the antitumor and antiviral capabilities of T cells (59), and whether perforin levels are altered in elderly CAP patients, thus influencing the severity of the disease, still requires further research. In summary, the changes in these biological markers provide important references for clinical practice and suggest that enhancing the application of biological markers in assessing the severity of CAP in elderly patients holds significant potential value. Therefore, future research can focus on regulatory T cells and their associated factors to provide more reliable biological evidence for assessing the severity of CAP in elderly patients. Additionally, research has shown that the number of early differentiated CD28+CD27+ T cells also has potential clinical significance in identifying high-risk elderly patients (15). CD28 and CD27 are costimulatory molecules necessary for T-cell activation and survival, and their fluctuations reflect changes in T-cell proliferative capacity (60). Changes in the proliferative capacity of T cells hold substantial significance for predicting disease prognosis in elderly patients with CAP.

## Frailty-related indicators

Compared with that among children and younger patients, frailty is more prevalent among elderly patients with CAP and is closely associated with increased mortality and prolonged hospital stays (61, 62). This phenomenon underscores the importance of a comprehensive assessment of frailty status in elderly CAP patients, highlighting the unique clinical significance of developing and validating relevant biological markers. Some studies have evaluated important biological markers for muscle mass, such as the ratio of aspartate aminotransferase to alanine aminotransferase (AST/ALT) and creatinine to cystatin C (Cr/CysC*100), and on the basis of their respective medians, elderly CAP patients are categorized into high- and low-muscle-mass groups (20). The results indicated that patients in the low-muscle-mass group had higher mortality rates at 1, 2, and 3 years than did those in the high-muscle-mass group did, further supporting the critical role of muscle mass in the prognosis of elderly CAP patients. Furthermore, the area of the Erector spinae muscle (ESM), an objective measure of muscle quality, has attracted researchers’ interest (63). Yoshikawa et al. (21) employed chest computed tomography (CT) to measure the cross-sectional area of the Erector spinae (ESMcsa), specifically obtaining the ESM cross-sectional area from single-row CT images at the level of the 12th thoracic vertebra and adjusting for body surface area (BSA). These findings demonstrated that lower ESMcsa/BSA values could effectively predict the 30-day mortality of elderly CAP patients, providing clinicians with a powerful prognostic assessment tool. Further studies indicate that the multidimensional prognostic index (MPI) is a validated prognostic tool based on the CGA, which can effectively predict the prognosis of elderly patients across various disease states (64–66). In recent research, the MPI, when used in combination with other biological markers, such as PCT and pro- adrenomedullin (Pro-ADM), has significantly improved the accuracy of mortality risk predictions for elderly CAP patients. In summary, through the assessment of biological markers related to frailty and the application of the MPI, we can more accurately predict the clinical outcomes of elderly patients with CAP, thereby providing crucial evidence for personalized treatment and clinical decision-making. These findings not only enhance our understanding of frailty in elderly CAP patients but also offer new perspectives and insights for future studies on biological markers (18, 19).

## Clinical value of other serum biological indicators in geriatric patients with CAP

Owing to the limitations inherent in prior etiological approaches, earlier studies have evaluated only a restricted range of agents or employed techniques of insufficient sensitivity. The predominant focus of investigations assessing the role of biomarkers in elucidating the etiology of CAP has been on the biomarker PCT (67–71). While many researchers acknowledge that the available data regarding the diagnostic efficacy of PCT for mixed infections remain limited, it is considered potentially useful for ruling out such infections (72, 73). In the context of viral versus bacterial CAP, studies have demonstrated that PCT has a high negative predictive value (NPV) for excluding bacterial respiratory mixed infections (74). Recently, as the understanding of CAP in the elderly population has considerably advanced, several novel biomarkers have attracted significant interest from the research community.

The clinical usefulness of C-reactive protein (CRP) in predicting prognosis and assessing the severity of CAP in elderly patients is mainly due to its role as an indicator of inflammation and its connection to the body’s systemic inflammatory response. CRP is produced by the liver when inflammation or infection is present; therefore, higher levels often reflect the body’s reaction to an infection (75). Research indicates that elevated CRP levels tend to correlate with the severity of the infection. CRP not only reflects the extent of the infection but may also be linked to systemic inflammatory response syndrome (SIRS) resulting from pneumonia (76). High levels of CRP are often indicative of activation of the inflammatory response, which may lead to organ function impairment and an increased risk of complications (77, 78). This is especially important in older patients who are more susceptible to multiple organ dysfunction. Measuring CRP can help doctors assess a patient’s inflammatory state and determine the severity of the disease.

In patients diagnosed with CAP, elevated levels of BNP frequently indicate underlying cardiac insufficiency or cardiac stress (79). The inflammatory response elicited by CAP can substantially augment the cardiac workload, particularly in individuals with preexisting cardiovascular comorbidities (80). Factors such as low blood oxygen and the release of inflammatory substances during pneumonia can further strain the heart, leading to increased BNP production (81). Studies have demonstrated that BNP is a reliable biomarker not just for the systemic inflammation related to pneumonia, but also for quantifying the heart’s burden and predicting risk in affected patients (82). Specifically, elevated BNP levels are correlated with increased mortality rates and a higher incidence of complications (82, 83). Consequently, BNP is a key indicator in clinical practice for assessing the prognosis of elderly patients with CAP (17). Moreover, cardiac insufficiency frequently precipitates pulmonary edema, which exacerbates dyspnea and compromises oxygenation, thereby adversely impacting overall patient prognosis. Therefore, the measurement of BNP levels empowers healthcare providers to devise more targeted and individualized therapeutic strategies, particularly in contexts necessitating concurrent management of cardiac pathologies.

Empirical studies have revealed a robust correlation between heightened D-dimer levels and both the severity and prognosis of CAP in elderly patients. Increased D-dimer concentrations are frequently interpreted as indicators of exacerbated disease severity and are correlated with elevated rates of mortality and associated complications (84). The underlying mechanisms of the prognostic utility and severity assessment of D-dimer in elderly individuals with CAP fundamentally pertain to its involvement in thrombotic mechanisms and inflammatory pathways. D-dimer is a byproduct of fibrin degradation; its elevated concentration typically signifies the activation of both coagulation and fibrinolytic pathways within the organism (85, 86). In the context of CAP, the systemic inflammatory response elicited by infection triggers the coagulation cascade, resulting in the formation of microthrombi. This process subsequently amplifies D-dimer production (87, 88). Specifically, inflammation from pneumonia damages and activates cells lining blood vessels (endothelial cells), which then release substances that promote clot formation. These microthrombi can impair the lungs’ ability to provide oxygen and raise the risk of serious complications, such as pulmonary embolism, worsening the patient’s condition. Consequently, elevated D-dimer acts as an important marker of this disease progression. Moreover, increased D-dimer levels are strongly linked to underlying conditions like heart disease and diabetes, which inherently increase the risk of blood clots in older adults. In these individuals, higher D-dimer levels not only indicate the severity of pneumonia but may also suggest a greater risk of death and complications (89, 90). Thus, D-dimer distinctly emerges as a crucial clinical indicator for the evaluation of prognosis and severity in elderly patients afflicted with CAP.

## Discussion

The diagnosis and prediction of CAP in elderly patients pose significant challenges, particularly in identifying uncertain cases. The symptoms of these cases frequently overlap with those of other conditions, making it difficult for clinicians to decide whether to pursue further testing or simply monitor the patient’s condition (91). This situation underscores the essential need for multidisciplinary collaboration, which can greatly enhance diagnostic accuracy.

This study assessed multiple biomarkers for managing CAP in older adults and generated several key observations. Overall, no reliable algorithm—based on a single marker or a panel—currently exists to define CAP etiology, and biomarkers have not demonstrated a clear advantage over conventional clinical methods for identifying specific pathogens. This limitation is amplified in elderly patients, whose pathogen spectrum is broad and whose rate of mixed infections is higher, making markers that indicate bacterial or mixed infection (notably PCT) of particular clinical interest. Given the ease of PCT measurement and the maturation of rapid bedside assays, its utility and clinical applications in older populations warrant continued evaluation (92).

Nevertheless, several unresolved issues temper enthusiasm for PCT and other emerging biomarkers in the elderly. First, the optimal diagnostic thresholds remain uncertain: multimorbidity, impaired renal function, and chronic inflammatory states common in older adults can shift biomarker baselines and may require subgroup-specific cutoffs. Second, the ideal timing for sequential monitoring is unclear; studies are needed to define when to sample and which temporal patterns most reliably reflect disease trajectory. Third, interpretative challenges persist—especially in patients with multiple comorbidities, malnutrition, or frailty—because the clinical meaning of rises or falls in a single biomarker can be ambiguous in these contexts. Addressing these gaps will require subgroup-stratified studies, standardized sampling protocols, and integration of biomarker data with host factors to improve diagnostic and prognostic utility in elderly CAP.

Managing pneumonia in advanced cancer patients presents unique complexities due to disease-related immunosuppression and the impact of treatments like chemotherapy and radiation (93). Consequently, personalized management strategies are paramount, requiring a comprehensive evaluation of each patient’s prognosis and treatment objectives. Concurrently, the escalating issue of antibiotic resistance poses a significant threat to high-risk groups, underscoring the importance of palliative care. A thorough understanding of prognostic indicators is crucial for guiding effective treatment decisions in this patient population. Moreover, vaccination remains a cornerstone in preventing infectious diseases among vulnerable individuals, necessitating rigorous assessments of vaccine safety and efficacy across diverse demographics, including the elderly and immunocompromised (94). Effective public health communication is also essential to improve vaccination rates, especially amidst widespread misinformation. Therefore, discussions on maintaining long-term immunity and the need for booster doses are increasingly pertinent.

The ongoing COVID-19 pandemic continues to challenge healthcare systems with concerns about vaccine effectiveness and the emergence of novel variants (95). Monitoring and tracking these variants, coupled with the development of appropriate public health responses and vaccination strategies, are critical. Global vaccination initiatives are vital to curbing the emergence of new variants, particularly as we prepare for seasonal epidemics akin to influenza. Finally, the phenomenon of long COVID encompasses a spectrum of persistent symptoms following acute infection, thereby complicating recovery and care pathways (96, 97). A thorough understanding of the pathophysiology of long COVID and its varied presentations requires interdisciplinary research to facilitate integrated patient care. This approach should be supported by effective management strategies, including rehabilitation and mental health services, to address these long-term sequelae. Sustained research into effective interventions for individuals experiencing long-term complications is indispensable. In conclusion, managing community-acquired pneumonia in the elderly requires careful attention to the potential of biomarkers, the importance of interdisciplinary collaboration, and the necessity of tailored treatment approaches. These efforts can enhance diagnostic precision and deliver comprehensive support, enabling patients to better navigate these complex medical challenges.

From a pathophysiological perspective, immunosenescence, accompanied by chronic low-grade inflammation (inflammaging), provides the biological foundation for the aberrant expression and interpretation of biomarkers in older adults (98). With aging, diminished CD28 expression on T cells, impaired costimulatory signaling, and the activation of inhibitory pathways such as PD-1 reshape immune responses, thereby influencing the production and temporal dynamics of inflammatory and immune markers (99). These immunological changes highlight the limitations of relying on “superficial” inflammatory markers and suggest the need for integrated biomarker panels that better reflect immune function and host physiological reserve (100, 101). Such panels—encompassing costimulatory/inhibitory profiles, lymphocyte functionality, and composite immune–inflammatory indices—should be validated across heterogeneous elderly populations to improve diagnostic precision, disease monitoring, and prognostic assessment.

In elderly patients with CAP, biomarker interpretation is particularly complex due to pronounced host heterogeneity, reflected in the wide variability of immune competence and anti-infective capacity. Consequently, the diagnostic or prognostic utility of individual biomarkers may not be consistent across all patients. Two interconnected mechanisms underlie this variability. First, age-related immunological alterations attenuate adaptive and innate immunity, manifested as weakened T-cell activation, reduced neutrophil chemotaxis and phagocytosis, and persistently elevated levels of cytokines such as IL-6 and TNF-α (102, 103). These processes interact synergistically: while impaired immunity hinders effective pathogen clearance, an elevated inflammatory baseline reduces the specificity of conventional markers, producing false positives or concealing the true severity of infection. Second, the burden of multimorbidity and reduced physiological reserve further amplifies biomarker variability.

Chronic diseases, malnutrition, and frailty alter both baseline levels (for example, renal disease elevates creatinine, hepatic dysfunction reduces albumin) and the kinetic behavior of markers during illness (104). Notably, frailty syndrome, as an integrated manifestation of diminished physiological reserve, independently modulates infection tolerance, disease progression, and biomarker dynamics (105). The convergence of immunosenescence, inflammaging, and frailty collectively weakens the discriminatory capacity of single biomarkers for etiological identification and prognostic prediction, as these signals are easily masked or confounded by the host’s physiological background. Therefore, in both research and clinical practice, an overreliance on isolated biomarkers should be avoided. Instead, multidimensional assessment frameworks that integrate clinical, functional, and biological dimensions—such as comorbidities, nutritional and frailty status, and host immune profiles—are essential for improving the accuracy of diagnosis, risk stratification, and therapeutic decision-making in elderly CAP.

Mechanistically, immunosenescence is characterized by thymic involution, reduced immune cell numbers, disrupted signaling pathways, and impaired cytokine secretion (103, 106, 107). In this context, costimulatory molecules (CSMs) play a vital role in T-cell activation and effector maintenance. Representative CSMs such as CD28, CD40L, OX40, and 4-1BB amplify T-cell responses through interactions with ligands including B7-1/B7-2, CD40, and GITR (108). Aging often results in decreased expression and function of these molecules—most notably CD28—leading to diminished cytokine production and weakened responses to infection and vaccination (109–111). Concurrently, persistent inflammation mediated by IL-6 and TNF-α activates inhibitory pathways such as PD-1, forming a “dual hit” of low costimulation and excessive inhibition that markedly impairs immune resilience in the elderly (112, 113).

Building upon these mechanisms, future studies should focus on identifying and validating biomarkers that reflect immune competence and host reserve, including costimulatory/inhibitory molecule expression patterns, immune cell phenotypes, and composite immune–inflammatory signatures, while also capturing their dynamic evolution throughout the disease course. Such mechanistic approaches may provide deeper insight into infection susceptibility and disease progression in older adults, as well as a biological basis for personalized interventions, such as modulation of costimulatory signaling or targeted reversal of immune dysfunction. Ultimately, these strategies aim to strengthen infection resistance and improve clinical outcomes for elderly patients with CAP.

The systematic influence of host heterogeneity on biomarker interpretation and predictive performance necessitates a paradigm shift in clinical and research practice: from dependence on isolated indicators toward multimodal, host-centered integrative assessment. We propose a structured framework that synthesizes five complementary information domains:(1) Conventional inflammatory and infection markers (CRP, PCT, leukocyte count, lactate) to detect infection presence and temporal evolution;(2) Nutritional, metabolic, and organ-function parameters (prealbumin/albumin, creatinine/eGFR, liver enzymes, glucose, electrolytes), reflecting metabolic reserve and organ dysfunction risk;(3) Immune function and host-reserve biomarkers—prioritized as a critical research avenue—encompassing lymphocyte subsets, costimulatory/inhibitory molecule expression profiles, suPAR, and syndecan-4, which quantify immunosenescence and infection tolerance;(4) Functional and frailty metrics (handgrip strength, gait speed, short frailty scales, Activities of Daily Living), capturing physiological reserve and recovery capacity;(5) Clinical severity scores and bedside parameters (CURB-65, PSI with geriatric modifications, vital signs, imaging-based assessments), enabling real-time triage. Horizontal integration of these domains with longitudinal tracking—at admission, 24–72 h, clinical stabilization/pre-discharge, and 30-/90-day intervals—simultaneously captures baseline host phenotype and dynamic disease progression, thereby identifying early “high-risk transition points” and defining critical treatment windows for precision intervention.

Evidence from recent multimodal studies underscores the clinical utility of this approach. For example, adding prealbumin to the PSI model enhanced mortality prediction sensitivity from 74.8% to 95.2% (114); incorporating suPAR and syndecan-4 further improved the AUC to 0.885 (115), demonstrating that immune and endothelial markers substantially augment risk stratification. However, these promising findings predominantly emerge from single-center or limited cohort designs, with insufficient validation in elderly subgroups characterized by multimorbidity and frailty—populations for whom predictive accuracy is most critical. To bridge this evidence gap, future research must prioritize: Multicenter, large-scale model development and external validation incorporating sequential, multi-timepoint biosampling to characterize marker kinetics, delineate optimal prediction windows, and establish dynamic decision thresholds. Model construction should integrate traditional statistical methods (mixed-effects modeling, survival analysis) with machine-learning approaches (LASSO regularization, ensemble methods, explainable AI), followed by rigorous calibration and decision-curve analysis in independent external cohorts. Stratified threshold validation across heterogeneous comorbidity profiles, renal function strata, and frailty grades is essential to minimize misclassification risk in vulnerable subpopulations.

Parallel translational development of clinician-accessible decision-support tools that visualize multimodal risk profiles and intervention pathways within electronic medical record systems. Pilot studies in diverse healthcare settings—from primary care to tertiary centers—should evaluate implementation feasibility, impact on antibiotic stewardship metrics, hospital length of stay, ICU admission rates, and 30-/90-day mortality. Concurrent biomarker-stratified randomized controlled trials and mechanistic substudies testing host-guided interventions—including biomarker-directed antibiotic de-escalation, targeted immunomodulation for immune dysfunction, and intensified nutrition/rehabilitation protocols—to establish causal links between multimodal host assessment and clinical outcomes or resource utilization. Rigorous attention to methodological and ethical safeguards, including: (i) standardized assay platforms with centralized quality control to minimize inter-site variability; (ii) age- and comorbidity-specific threshold calibration guidelines; (iii) secure proxy consent mechanisms for cognitively impaired patients; and (iv) health economic analyses examining cost-effectiveness and accessibility in resource-constrained settings. Through this comprehensive pathway—combining mechanistic insights, robust validation, pragmatic implementation, and rigorous governance—a biomarker-informed, host-centric model can maximize diagnostic and prognostic precision while honoring the inherent physiological heterogeneity of older adults, ultimately advancing CAP management toward evidence-based personalization, clinical efficiency, and equitable resource stewardship.

Although this article provides a comprehensive discussion of biomarkers in elderly patients with CAP, it has several limitations. First, it does not cover all potential biomarkers related to CAP, such as myristoyl lysophosphatidylcholine (116), serum neurofilament light chain (117), complement C3a (118), serum survivin (119), serum TRAIL (120), N-myc and STAT interaction protein (121), serum IL-2 (122), serum IL-27 (123), surfactant protein D, human cartilage glycoprotein YKL-40, and chemokine ligand 18 (124), and fibroblast growth factor 21 (125),as well as markers of neutrophil extracellular traps (126). Additionally, cardiac-related biomarkers were not included in the discussion (127). These biomarkers may hold significant clinical relevance in the management of elderly patients with CAP, and there has been no discussion regarding the role of biomarkers in the treatment of elderly patients with CAP, particularly concerning the initiation, continuation, and duration of antibiotic therapy, where advances have been made. PCT has been extensively evaluated as a biomarker to guide antibiotic therapy in suspected bacterial infections. In several randomized trials and pragmatic studies conducted in predominantly non-geriatric cohorts, PCT-guided algorithms have been associated with reductions in total antibiotic exposure and antibiotic duration without a concomitant increase in adverse clinical outcomes, thereby contributing to antimicrobial stewardship and mitigation of drug-related harms (128–130).

However, the use of PCT as a solitary decision-making instrument in elderly patients with CAP warrants considerable caution. Critical parameters — including age-specific optimal thresholds, the temporal kinetics of PCT during the disease course in older adults, and the modulatory effects of prevalent geriatric factors (multimorbidity, chronic organ dysfunction, pharmacologic immunosuppression and frailty) — remain insufficiently characterized. These conditions may independently alter baseline PCT concentrations or its inducibility in response to infection, thereby complicating interpretation and potentially reducing diagnostic and prognostic accuracy. Accordingly, in geriatric clinical practice biomarkers should not supplant comprehensive clinical assessment but rather be integrated into a multimodal decision framework that encompasses hemodynamic and respiratory vital signs, imaging findings, trajectory of signs and symptoms, and microbiological data (e.g., sputum/urine cultures), together with formal appraisal of functional status and frailty. Practical application strategies may include the following: (1) Antibiotic initiation: In elderly patients who present with relatively mild clinical signs but demonstrate a rapid and sustained rise in PCT together with compatible clinical features of bacterial infection, early initiation or continuation of empirical antibiotic therapy may be justified, with intensification of clinical surveillance and serial biomarker measurement. This approach is particularly pertinent in frail individuals or those with diminished physiological reserve, in whom delays in therapy may lead to worse outcomes. (2) Antibiotic duration and de-escalation: For elderly patients who are clinically improving, and in whom PCT levels remain persistently low or show a clear downward trajectory in conjunction with reassuring clinical and microbiological data, consideration may be given to shortening the antibiotic course.

This study has several important limitations that should be considered when interpreting the findings. First, publication bias may have influenced the available evidence: studies reporting statistically significant or positive results are more likely to be published, which may lead to an overestimation of the clinical utility of certain biomarkers. Second, substantial heterogeneity across the included studies limits comparability and generalizability. Heterogeneity was evident in study settings (outpatient, general ward, ICU), definitions of older age (e.g., ≥ 65, ≥ 75 years), comorbidity burden and profiles, prior antimicrobial exposure, methods of pathogen ascertainment (culture vs. molecular diagnostics), and outcome definitions and follow-up intervals. These sources of variability can materially affect biomarker baseline levels, dynamic responses, and their diagnostic and prognostic performance. Third, many primary studies lacked preplanned or adequately powered subgroup analyses within elderly strata (for example, 65–74, 75–84, ≥ 85 years) or by frailty status, hampering precise extrapolation of results to older or severely frail subpopulations. Fourth, the presentation of diagnostic performance metrics in this review is uneven across biomarkers. This disparity reflects several factors rather than implying absence of evidence or clinical irrelevance for markers lacking full metrics: (1) availability of evidence — well-studied markers (e.g., PCT, CRP) have multiple high-quality diagnostic studies and meta-analyses, whereas emerging markers are often reported only in preliminary or correlation studies without formal accuracy assessments; (2) methodological heterogeneity — differences in reference standards, cutoff selection, and patient populations preclude meaningful pooling or direct comparison for some markers, so we prioritized qualitative description of biological plausibility and clinical associations over reporting single, potentially misleading point estimates; (3) differing intended applications — certain markers are investigated primarily for prognostication or disease monitoring (reporting hazard ratios or longitudinal trends) rather than initial diagnostic accuracy; and (4) paucity of elderly-specific data — many studies did not provide age-stratified results, limiting the ability to report elderly-specific performance metrics. We acknowledge that this inconsistent reporting could be misinterpreted as privileging biomarkers with complete numerical metrics. To avoid this misperception, we emphasize that the heterogeneity in data completeness reflects current research gaps and focus areas, not an *a priori* assessment of marker validity. Finally, most evidence originates from single-center or single-cohort investigations, with relatively few large-scale, multicenter, multiethnic validation studies; this constrains the external validity of our conclusions across different healthcare systems and patient populations. Future research should prioritize standardized outcome definitions, age- and frailty-stratified analyses, and prospective, multicenter validation of promising biomarker panels.

In summary, this study emphasizes the important value of biomarkers in the etiological diagnosis, disease assessment, and prognostic prediction of elderly patients with CAP. Clinicians can utilize these biomarkers for a comprehensive evaluation of elderly CAP patients to formulate individualized treatment plans, optimize management strategies, and improve patient outcomes. Future research should continue to explore other potential biomarkers and their applications in different clinical contexts to provide a more comprehensive basis for improving the prognosis of elderly CAP patients.
