# Supplementary material for: Potential role of serological biomarkers in the diagnosis and prediction of community-acquired pneumonia in elderly individuals
Source: Front Med (Lausanne). 2025 Nov 20;12:1699779. doi: 10.3389/fmed.2025.1699779 (PMC12675376; doi:10.3389/fmed.2025.1699779)
Supplement: Supplementary file 1 [file Data_Sheet_1.pdf]

Appendix S1: Example PubMed Search Strategy

This appendix details the search strategy employed in the PubMed database to comprehensively identify relevant literature on the application of biomarkers in diagnosis and prognosis for elderly patients with community-acquired pneumonia. The search strategy integrates PubMed Medical Subject Headings (MeSH Terms) and free-text terms (Title/Abstract), combining the following four core concepts through Boolean operators (AND, OR).

Search Period: 2010–2025

Core Concept 1: Community-Acquired Pneumonia (CAP)

Type	Search Terms
MeSH Terms	"Pneumonia, Community-Acquired"[MeSH Terms]
Free Text Terms	"community acquired pneumonia"[Title/Abstract]<br>CAP[Title/Abstract]

Logical Combination:

(("Pneumonia, Community-Acquired"[MeSH Terms]) OR ("community acquired pneumonia"[Title/Abstract])) OR (CAP[Title/Abstract])

Core Concept 2: Biomarkers

Type	Search Terms
MeSH Terms	"Biomarkers"[MeSH Terms]
Free Text Terms	"serological biomarkers"[Title/Abstract]<br>"serum biomarkers"[Title/Abstract]<br>"inflammatory markers"[Title/Abstract]<br>"procalcitonin" [Title/Abstract]<br>"C-reactive protein"[Title/Abstract]<br>"CRP" [Title/Abstract]<br>"cytokines" [Title/Abstract]

Logical Combination:

(("Biomarkers"[MeSH Terms]) OR ("serological biomarkers"[Title/Abstract])) OR ("serum biomarkers"[Title/Abstract]) OR ("inflammatory markers"[Title/Abstract]) OR (procalcitonin[Title/Abstract]) OR ("C-reactive protein"[Title/Abstract]) OR (CRP[Title/Abstract]) OR (cytokines[Title/Abstract])

Core Concept 3: Aged (Older Adults)

Type	Search Terms
MeSH Terms	" Aged "[MeSH Terms]
Free Text Terms	"elderly" [Title/Abstract]<br>"older adults"[Title/Abstract]<br>"geriatric" [Title/Abstract]<br>"aged 65"[Title/Abstract]

Logical Combination:

(("Aged"[MeSH Terms]) OR (elderly[Title/Abstract])) OR ("older adults"[Title/Abstract]) OR (geriatric[Title/Abstract]) OR ("aged 65"[Title/Abstract])

Core Concept 4: Diagnosis/Prognosis/Severity

Type	Search Terms
MeSH Terms	"Diagnosis"[MeSH Terms]<br>"Prognosis"[MeSH Terms]
Free Text Terms	"diagnostic" [Title/Abstract]<br>"prognostic" [Title/Abstract]<br>"prediction" [Title/Abstract]<br>"severity" [Title/Abstract]

Logical Combination:

(("Diagnosis"[MeSH Terms]) OR ("Prognosis"[MeSH Terms])) OR (diagnostic[Title/Abstract]) OR (prognostic[Title/Abstract]) OR (prediction[Title/Abstract]) OR (severity[Title/Abstract])

Final PubMed search:

The following is the final search formula formed by connecting the four core concepts above using the AND logical operator, and is used to perform the search in the PubMed database:

(((("Pneumonia, Community-Acquired"[MeSH Terms]) OR ("community acquired pneumonia"[Title/Abstract])) OR (CAP[Title/Abstract])) AND (((("Biomarkers"[MeSH Terms]) OR ("serological biomarkers"[Title/Abstract])) OR ("serum biomarkers"[Title/Abstract])) OR ("inflammatory markers"[Title/Abstract]) OR (procalcitonin[Title/Abstract]) OR ("C-reactive protein"[Title/Abstract]) OR (CRP[Title/Abstract]) OR (cytokines[Title/Abstract]))) AND (((("Aged"[MeSH Terms]) OR (elderly[Title/Abstract])) OR ("older adults"[Title/Abstract])) OR (geriatric[Title/Abstract]) OR ("aged 65"[Title/Abstract]))) AND (((("Diagnosis"[MeSH Terms]) OR ("Prognosis"[MeSH Terms])) OR (diagnostic[Title/Abstract])) OR (prognostic[Title/Abstract]) OR (prediction[Title/Abstract]) OR (severity[Title/Abstract])))
